# Supplementary material for: Interplay of p23 with FKBP51 and their chaperone complex in regulating tau aggregation
Source: Nat Commun. 2025 Jan 14;16:669. doi: 10.1038/s41467-025-56028-0 (PMC11733250; doi:10.1038/s41467-025-56028-0)
Supplement: Supplementary file 1 — Supplementary Information [file 41467_2025_56028_MOESM1_ESM.pdf]

Supplementary information for

Interplay of p23 FKBP51 and their chaperone complex in regulating tau aggregation

Pijush Chakraborty[1], Markus Zweckstetter[1,2,*]

[1] Department for NMR-based Structural Biology, Max Planck Institute for Multidisciplinary Sciences, Am Faßberg 11, 37077, Göttingen, Germany.

[2] German Center for Neurodegenerative Diseases (DZNE), Von-Siebold-Str. 3a, 37075 Göttingen, Germany.

*Correspondence should be addressed to markus.zweckstetter@dzne.de

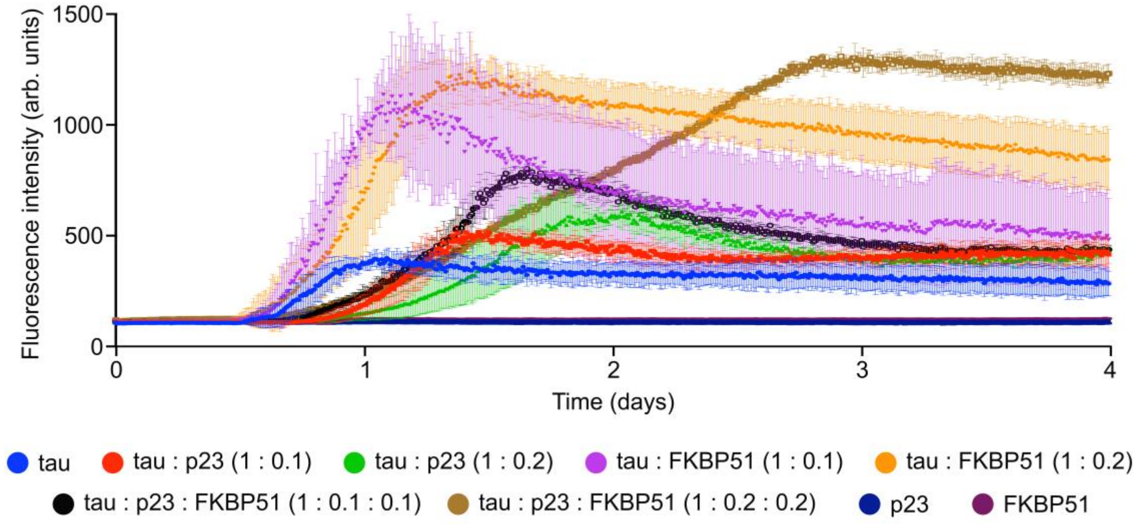

Supplementary Fig. 1 | p23-FKBP51 modulates the aggregation of tau. Aggregation kinetics of 25 µM tau in the absence or presence of different co-chaperones. Error bars represent the std of three independently aggregated samples. The center of the error bars represents the average value of three independent samples. Source data are provided as a Source Data file.

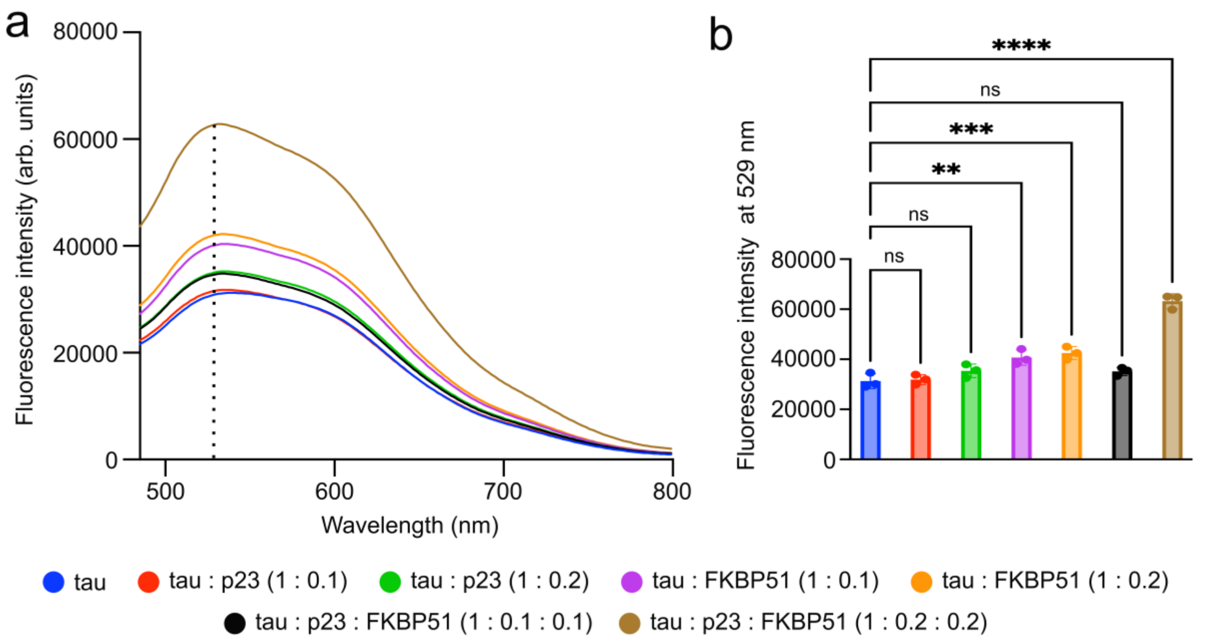

Supplementary Fig. 2 | Binding of fluorescent dye curcumin to the preformed tau fibril. a, Fluorescent emission spectra of amyloid-binding dye curcumin upon binding with different preformed tau fibrils. The fibrils were generated by the aggregation of tau in the absence or presence of different co-chaperones. The peak at 529 nm is represented by the dotted line. The average emission curves from three independent experiments are shown. **b**, Fluorescence intensity at the peak (529 nm) of curcumin when bound to different tau fibrils. Error bars represent the std of three independent experiments. The center of the error bars represents the average value of three independent experiments. Statistical analysis was performed using one-way ANOVA analysis. **$p = 0.0028$, ***$p = 0.0006$, ****$p < 0.0001$. Source data are provided as a Source Data file.

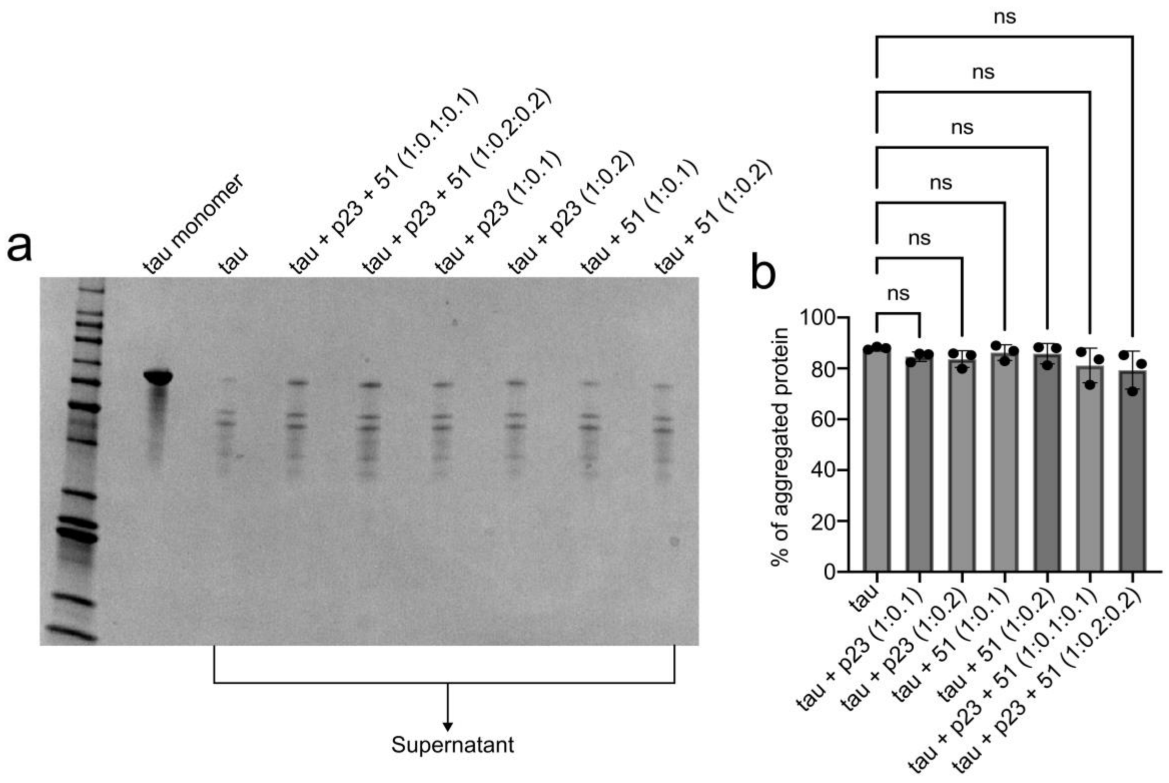

Supplementary Fig. 3 │ Determination of the amount of aggregated protein. a, A representative SDS-PAGE gel of tau monomer and supernatant (SN) (after pelleting down the fibrils) of tau either in the absence or presence of different co-chaperones. The fibril samples were collected after four days of aggregation. **b**, Calculation of the amount of aggregated protein. The amount of aggregated protein was calculated by comparing the intensity of the supernatant (SN) band to the tau monomer band. Error bars represent the std of three independent SDS-PAGE gels. The center of the error bars represents the average value of three independent experiments. Statistical analysis was performed using one-way ANOVA analysis. Source data are provided as a Source Data file.

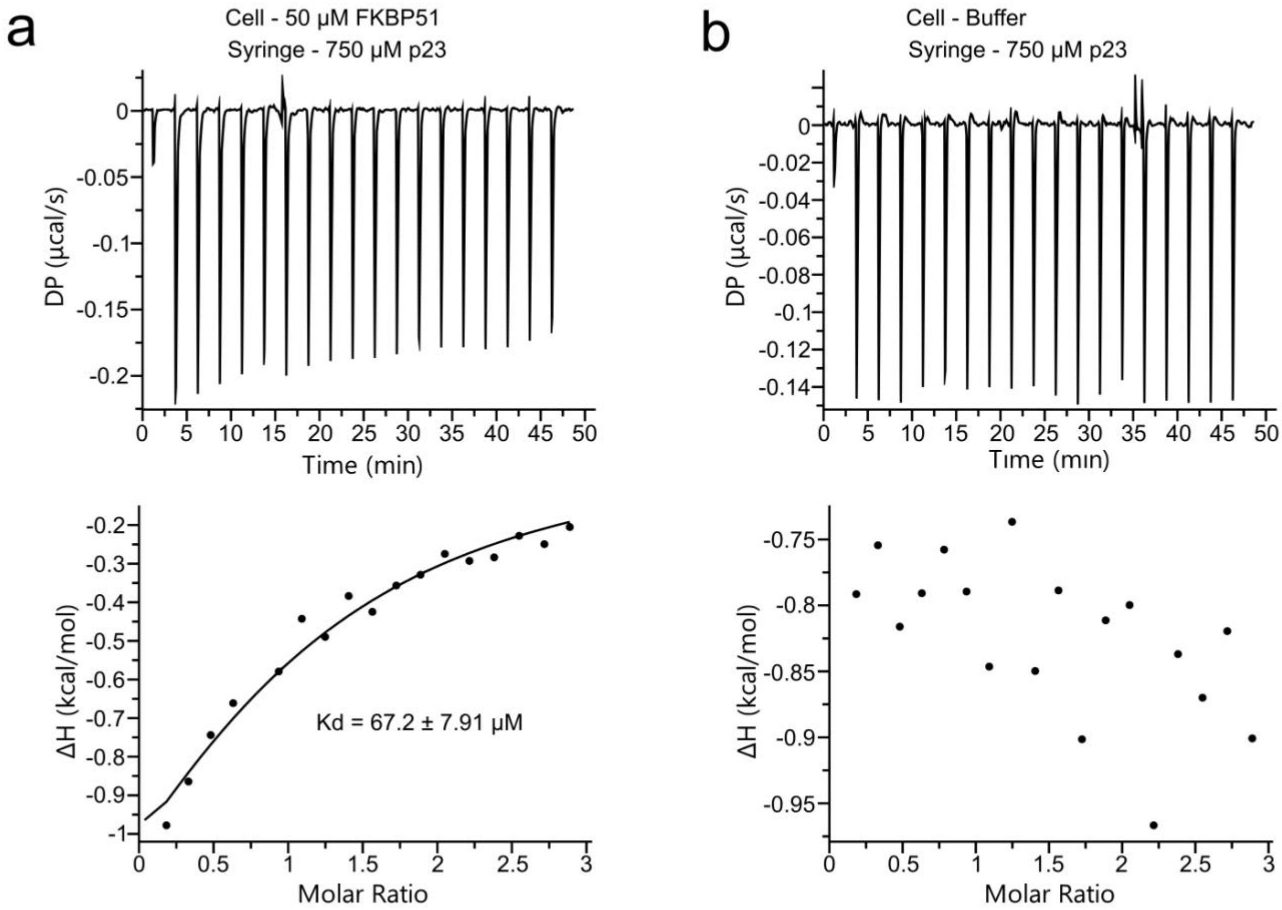

Supplementary Fig. 4│ Determination of the binding affinity between p23 and FKBP51. a, Isothermal titration calorimetry (ITC) experiment between p23 and FKBP51. The sample cell was loaded with 50 μM FKBP51 and titrated with 750 μM of p23 at 25 °C in 25 mM HEPES, 10 mM KCl, 5 mM MgCl2, and 1 mM DTT, pH 7.2. The data was fitted for one set of binding sites resulting in ΔH = -2.29 ± 0.125 kcal/mol, ΔG = -5.69 kcal/mol, and -TΔS = -3.41 kcal/mol. **b**, Control ITC experiment to account for the heat of dilution upon addition of 750 μM p23 to 25 mM HEPES, 10 mM KCl, 5 mM MgCl2, and 1 mM DTT, pH 7.2 buffer at 25 °C.

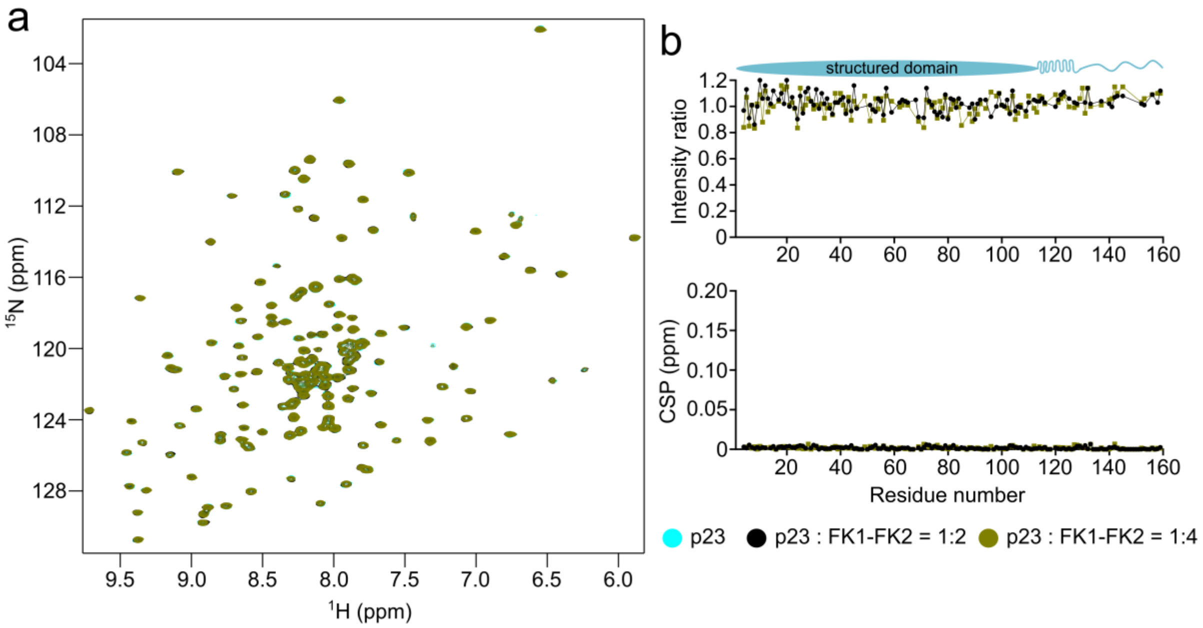

Supplementary Fig. 5 | Interaction of p23 with FK1-FK2. a, 2D ^{1}H-^{15}N TROSY HSQC spectra of ^{15}N-labeled p23 in the absence (cyan) or presence of two-fold (black) and four-fold (deep green) molar excess of unlabeled FK1-FK2. **b**, Changes in the intensities (top) and chemical shift perturbations (CSPs) (bottom) of the cross peaks in the TROSY-HSQC spectrum of p23 upon the addition of two-fold (black) and four-fold (deep green) molar excess of unlabeled FK1-FK2. The domain diagram of p23 is shown at the top. Source data are provided as a Source Data file.

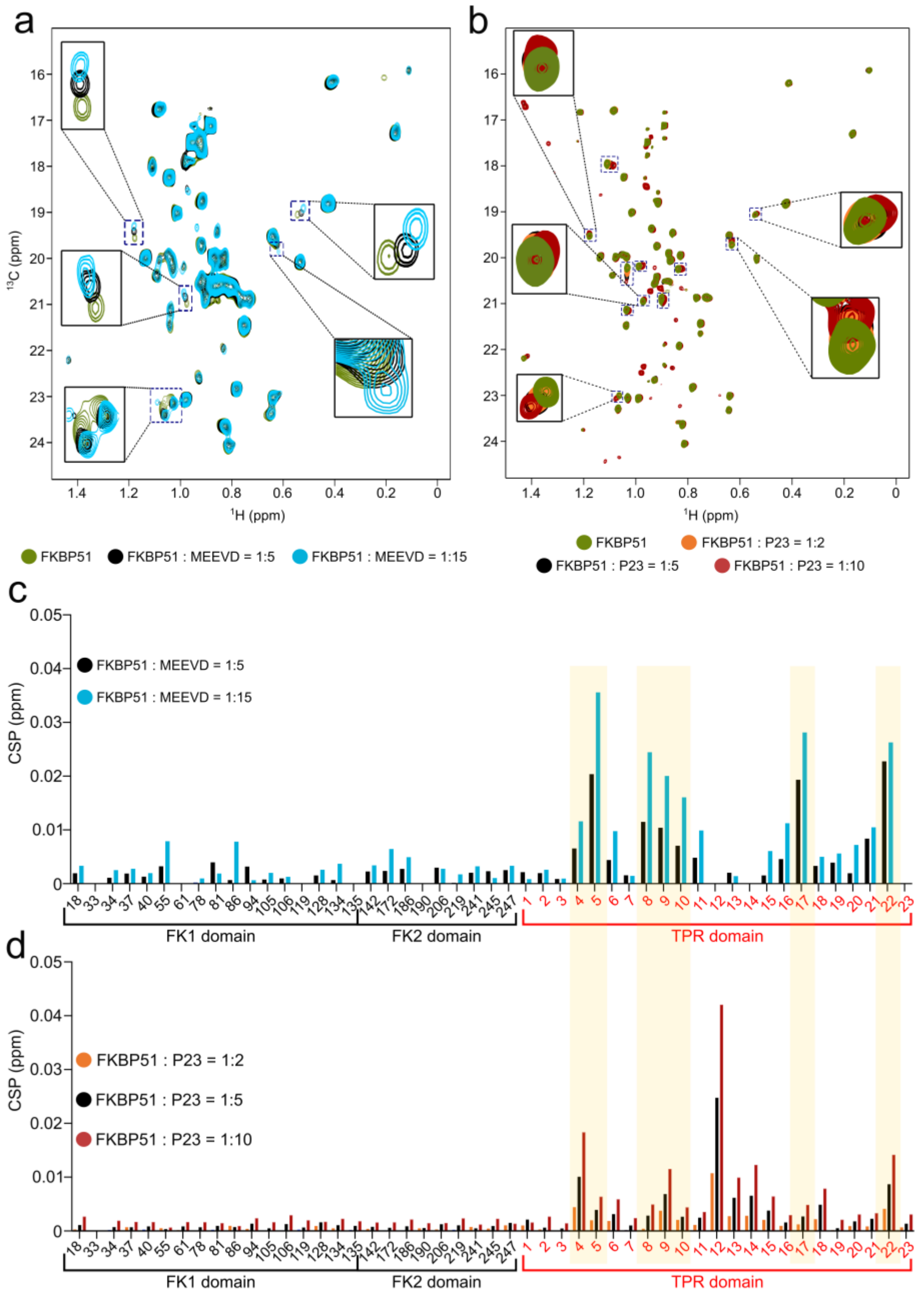

Supplementary Fig. 6 | Comparison of the binding site of p23 and MEEVD peptide on FKBP51. **a,** 2D ^{1}H-^{13}C methyl-TROSY spectra of ^{13}C-methyl labeled FKBP51 in the absence (green) or presence of five-fold (black) and fifteen-fold (sky blue) molar excesses of MEEVD peptide. The perturbed resonances from the TPR domain are zoomed in. **b**, 2D ^{1}H-^{13}C HMQC spectra of ^{13}C-methyl labeled FKBP51 in the absence (green) or presence of two-fold (orange), five-fold (black), and ten-fold (red) molar excesses of unlabeled p23. The perturbed resonances shown in (a) are also zoomed in. **c**, Chemical shift perturbations (CSP) of the cross peaks of the methyl groups of FKBP51 (a) upon addition of five-fold (black) and fifteen-fold (sky blue) molar excesses of MEEVD peptide. The CSPs of residues present in the TPR domain are indicated by peak numbers from 1 to 23 as shown in Fig. 3b. **d**, Chemical shift perturbations (CSP) of the cross peaks of the methyl groups of FKBP51 (b) upon addition of two-fold (orange), five-fold (black), and ten-fold (red) molar excess of unlabeled p23. The CSPs of residues present in the TPR domain are indicated by peak numbers from 1 to 23 as shown in Fig. 3b. The residues in the TPR domain of FKBP51 that were perturbed by both MEEVD and p23 are highlighted with yellow boxes. Source data are provided as a Source Data file.

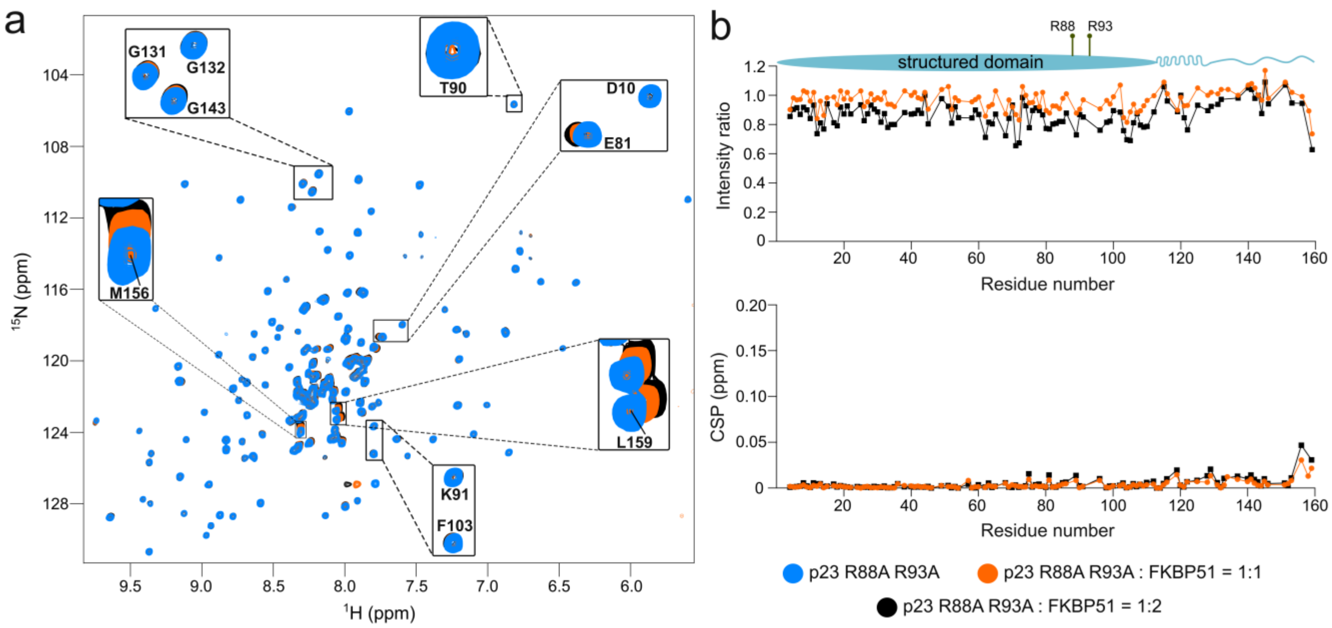

Supplementary Fig. 7 | Interaction of R88A/R93A-mutant p23 with FKBP51. a, 2D ^{1}H-^{15}N TROSY HSQC spectra of deuterated, ^{15}N-labeled R88A/R93A-mutant p23 either in the absence (blue) or presence of equimolar (orange) and two-fold (black) molar excess of unlabeled FKBP51. **b**, Changes in the intensities (top) and chemical shift perturbations (CSPs) (bottom) of the cross peaks in the TROSY-HSQC spectrum of R88A/R93A-mutant p23 upon the addition of equimolar (orange) and two-fold (black) molar excess of FKBP51. The domain diagram of p23 with the positions of R88 and R93 is shown above. Source data are provided as a Source Data file.

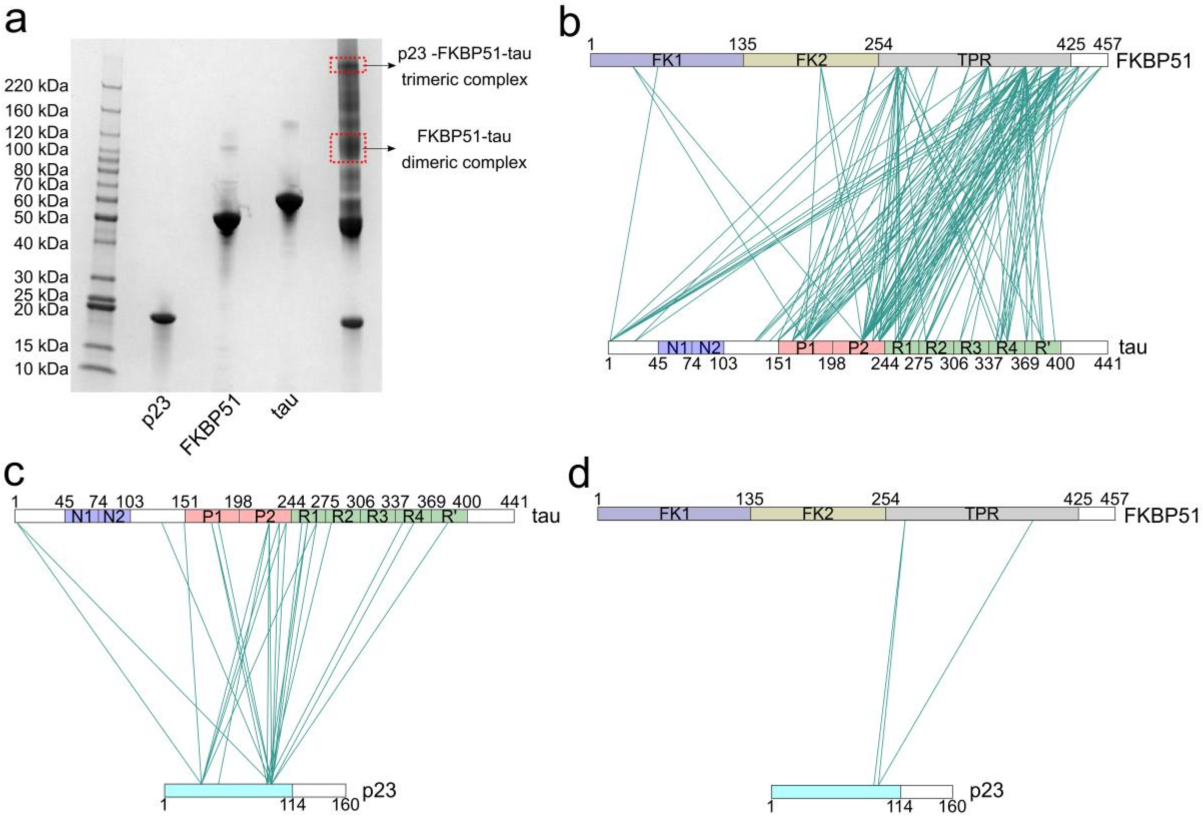

Supplementary Fig. 8 | Experimental validation of p23-FKBP51-tau trimeric complex formation. **a,** SDS-PAGE gel of p23, FKBP51, tau, and DSS-crosslinked p23-FKBP51-tau trimeric complex. The bands of the trimeric complex and FKBP51-tau dimeric complex analyzed by mass spectrometry are highlighted by a red dotted box. **b,c,d,** Intermolecular crosslinks between FKBP51, p23, and tau. The details of the crosslinked residues are shown in Supplementary Data 1. Source data are provided as a Source Data file.

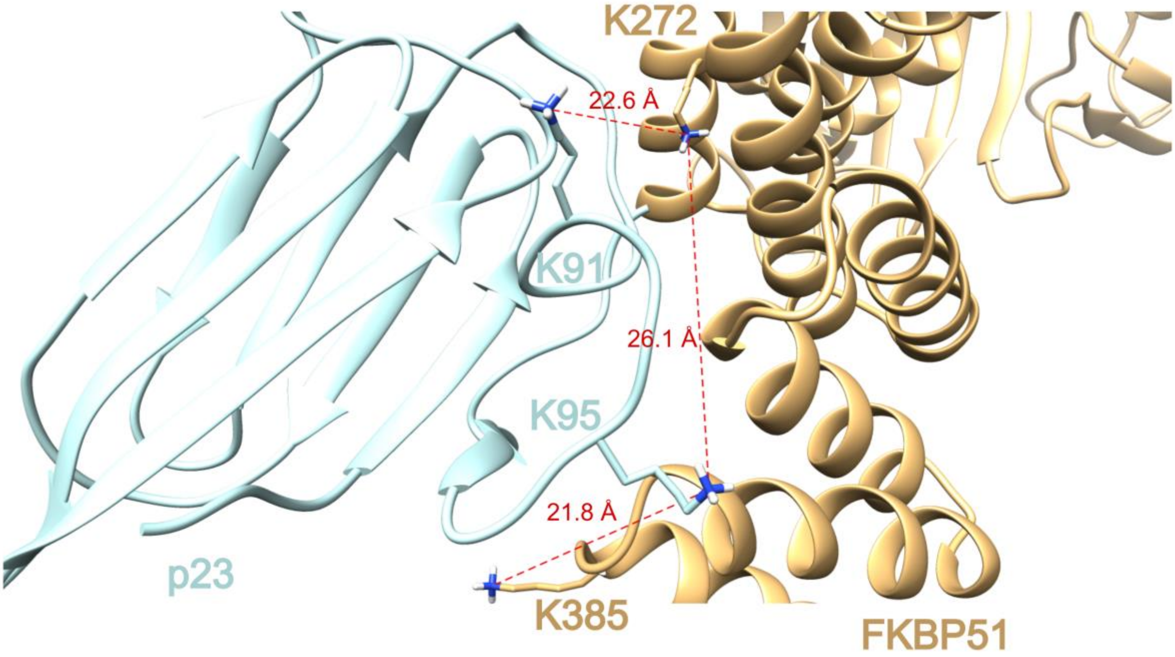

Supplementary Fig. 9│ Mapping of the crosslinks on the structural model of p23-FKBP51 complex. The crosslinks observed between K91 (p23) – K272 (FKBP51), K95 (p23) – K272 (FKBP51), and K95 (p23) – K385 (FKBP51) are shown, and the distances between the Nz atoms of the lysine residues indicated.

HADDOCK score	-146.9 ± 3.4
Cluster size	92
RMSD from the lowest-energy structure	0.9 ± 0.5
Van der Waals energy	-44.2 ± 5.7
Electrostatic energy	-668.3 ± 41
Desolvation energy	27 ± 3.5
Buried surface area	2014.9 ± 115.2
Z-score	-1.9

Supplementary Table 1│ HADDOCK docking statistics of the structure of p23-FKBP51 complex

Mutant	Forward primer	Reverse primer	Template
p23 (1-119)	5´CAGATGAAGACATGTCTAATTAAGATCG TTTCTCTGAGATGATG 3´	5´CATCATCTCAGAGAAACGATCTTAA TTAGACATGTCTTCATCTG 3´	p23
p23 R88A	5´CAGTCATGGCCAGCGTTAACAAAAGAA AGG 3´	5´CCTTTCTTTTGTTAACGCTGGCCATG ACTG 3´	p23
p23 R88A R93A	5' CCAGCGTTAACAAAAGAAGCGGCAAAGC TTAATTGGC 3'	5' GCCAATTAAGCTTTGCCGCTTCTTTTG TTAACGCTGG 3'	p23 R88A

Supplementary Table 2 | Details of the primers to generate different mutants of p23
