# Supplementary material for: Interplay of p23 with FKBP51 and their chaperone complex in regulating tau aggregation
Source: Nat Commun. 2025 Jan 14;16:669. doi: 10.1038/s41467-025-56028-0 (PMC11733250; doi:10.1038/s41467-025-56028-0)
Supplement: Supplementary file 2 — Description of Additional Supplementary Information [file 41467_2025_56028_MOESM2_ESM.pdf]

Title: Supplementray Data 1

Description: Details of the intermolecular crosslinks detected between p23, FKBP51, and tau in the DSS-crosslinked complex. Band 1 refers to the upper band (trimeric complex) in the gel shown in Supplementary Fig. 8a & band 2 refers to the lower band (dimeric FKBP51-tau complex) in the gel shown in Supplementary Fig. 8a.
